# Single-Tunnel Double-Bundle-Like Effect With Footprint Enhancing Anterior Cruciate Ligament Reconstruction

**DOI:** 10.1016/j.eats.2021.10.023

**Published:** 2022-02-08

**Authors:** Mukesh S. Laddha, S.V. Gowtam, Prakhar Jain

**Affiliations:** RNH Hospital, Balraj Marg, Dhantoli, Nagpur, Maharashtra, India

## Abstract

The anterior cruciate ligament (ACL) consists of an anteromedial bundle and a posterolateral bundle giving anteroposterior and rotational stability. It’s one of the most commonly injured ligaments and also one of the most commonly performed arthroscopic procedures. Management of ACL injuries is one of the most frequently studied subjects in the literature. Surgical management of ACL injuries varies from extraarticular tenodesis to arthroscopic transtibial reconstruction to double-bundle reconstruction to anatomic single-bundle reconstruction. Although double-bundle ACL reconstruction gives more rotational stability than anatomic single-bundle, functional outcome of both are the same, but the complication rates are much higher for double-bundle reconstruction. Hence, anatomic single-bundle ACL reconstruction has gained popularity. The femoral and tibial footprint of the ACL varies in shape and size; it can be oval, elliptical, rectangular, C-shape, and more. But all available ACL reconstruction techniques prepare a circular tunnel; hence, the footprint coverage of the native ACL is maximum after double-bundle reconstruction and less after anatomic single-bundle reconstruction. So, to have the benefit of double-bundle reconstruction with a single tunnel, we propose our technique of a single-tunnel double-bundle-like effect, with the footprint enhancing ACL reconstruction using our newly designed tunnel dilators.

The anterior cruciate ligament (ACL) consists of 2 bundles: the anteromedial (AM) and the posterolateral (PL). Biomechanically, these 2 bundles function together to provide stability to the knee throughout the range of motion.^1^, ^2^, ^3^ The AM bundle is primarily responsible for stabilization of the knee in the anterior-posterior direction, whereas the PL bundle provides rotational stability.^1^, ^2^, ^3^, ^4^ The AM and PL bundles work in synchronization during knee movements. The AM bundle is tight in flexion, and the PL bundle is tight in extension.^5^ The incidence of ACL tear in the general population has been estimated to be between 30 to 78 per 100,000 people.^6^^,^^7^ The treatment of an ACL tear is one of the most frequently studied and updated treatments in the orthopaedic literature.^8^ It is notable that 80% of knee ligament surgeries involve ACL surgery.^9^

Techniques for ACL repair or reconstruction has been evolving. In 1967, Lemaire^10^^,^^11^ described an extra-articular reconstruction of a ruptured ACL by anterolateral (AL) tenodesis. Then Insall et al.^12^ introduced intra-articular reconstruction which laid foundation for transtibial technique. The arthroscopic transtibial ACL reconstruction technique had the drawback of tunnel malposition and rotational instability.^13^^,^^14^ Muneta et al.^15^ introduced the double-bundle technique, which had improved anterior laxity and rotational stability but had its own complications like abundant fixation, longer surgery, tunnel coalescence, difficult revision surgery, and high technical expertise.^16^ So anatomical-single-bundle reconstruction through the medial portal technique gained importance and had functional results similar to double-bundle reconstruction.^17^ AL ligament reconstruction and lateral extra-articular tenodesis gained importance to achieve more rotational stability in revision ACL reconstruction and primary ACL with grade III pivot shift.^18^

The footprint coverage in the single-bundle reconstruction technique is 57% of the native tibial insertion as mentioned by Kopf et al.^19^ and 61% of the femoral insertion as mentioned by Hensler et al.^20^ The double-bundle reconstruction technique has the potential to cover up to 97% or more of the footprint area.^21^ Anatomic studies of the ACL are of the suggestion that femoral ACL insertion is crescent shaped.^22^, ^23^, ^24^, ^25^, ^26^ In addition, it has been suggested that the ACL femoral footprint varies in shape and can be circular, elliptical, kidney shaped, trapezoidal, ovoid, or triangular.^27^ The tibial insertion site was found to be elliptical (51%), triangular (33%), and C-shaped (16%).^28^ All available single-bundle reconstruction techniques create a circular tunnel that covers only a portion of the anatomic footprint. Our technique covers the maximum available footprint in size and shape through a single oval femoral and rectangular tibial tunnel, thereby creating a double-bundle-like effect with single-bundle reconstruction.

## Surgical Technique

### Patient Positioning

**Fig 1:**
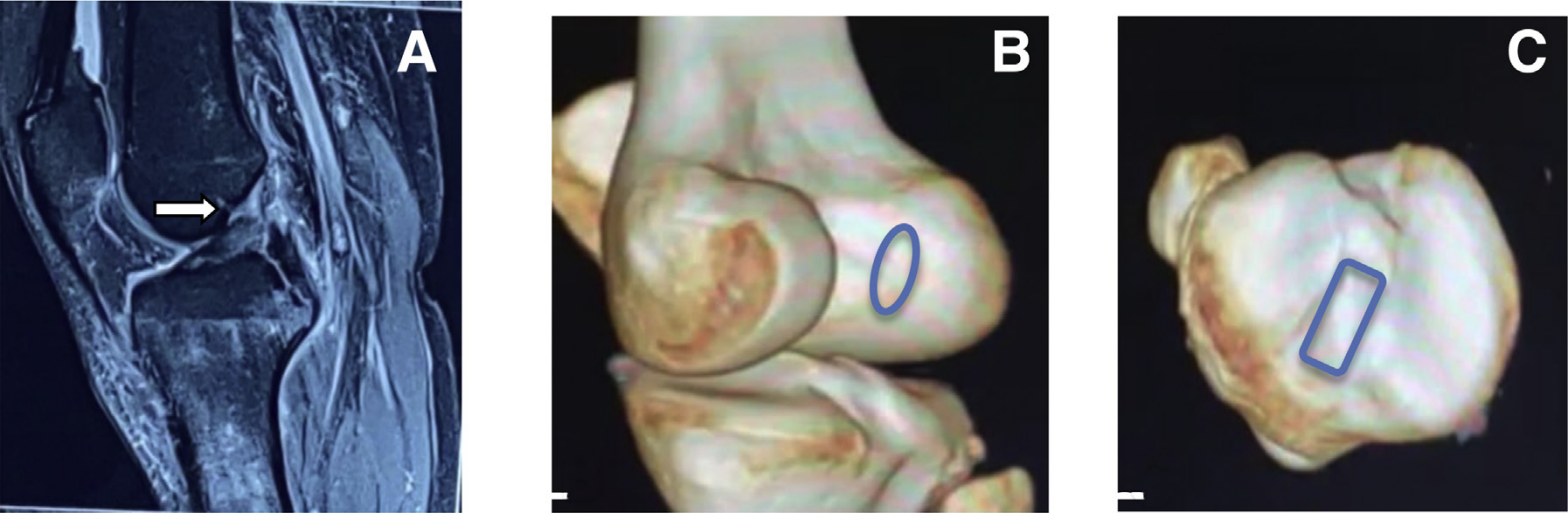
A young man with knee instability, clinically Lachman and anterior drawer grade III, suggestive of anterior cruciate ligament (ACL) injury. Preoperative MRI and 3D-CT showing complete full-thickness ACL tear from the femoral side and the footprint anatomy. (A) Sagittal PDFS MRI of right knee demonstrates ACL tear shown by a white arrow. (B) Preoperative 3D-CT scan of the right knee demonstrating an oval femoral footprint as marked by the blue oval. (C) Preoperative 3D-CT scan of the right knee demonstrating a rectangular tibial footprint as marked by the blue rectangle. PDFS, proton density fat suppressed; MRI, magnetic resonance imaging; 3D-CT, 3-dimensional computed tomography.

With the patient under spinal anesthesia, a pneumatic tourniquet cuff is applied with adequate padding to the proximal aspect of right thigh. The patient is placed in a supine position with the knee hanging 90° at the caudal end of the operating table. Lateral thigh support is attached to stabilize the knee during valgus stress force. Painting and draping are performed with all aseptic precautions. The tourniquet is inflated up to 300 mm Hg (Figure 1).

### Portal Placement

Standard high AL primary portal is placed just below the lower pole of patella and lateral to the patellar tendon. A second low AM horizontal portal is made under direct vision using long spinal needle to prevent iatrogenic injury to medial meniscus. Posteromedial (PM) portal prepared under vision using spinal needle 1 cm above the joint line and behind the MCL. The transpatellar (TP) portal is prepared around 1 cm distal to lower pole patella by splitting the tendon (Video 1).

**Fig 2:**
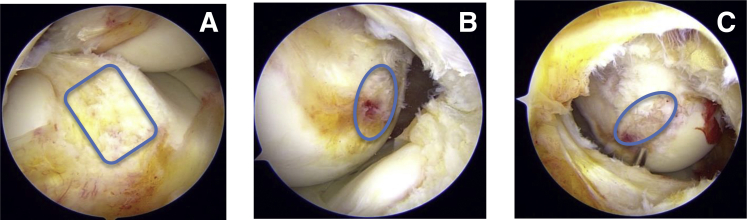
(A) Arthroscopic view from the high anterolateral portal showing the rectangular tibial footprint of the anterior cruciate ligament (ACL) as marked by a blue rectangle. (B) Arthroscopic view from the transpatellar portal showing oval femoral footprint of ACL as marked by a blue oval. (C) Arthroscopic view from the posteromedial portal showing the oval femoral footprint of the ACL as marked by a blue oval.

Diagnostic scopy revealed full-thickness ACL tear from the femoral side. Both medial and lateral meniscus, posterior cruciate ligament (PCL) and cartilage is intact. Complete debridement of ACL stump was is done and both tibial and femoral footprints are exposed. View from high AL portal clearly showed rectangular shaped ACL tibial footprint (Fig 2A). View from TP and PM portal showed oval femoral footprint (Fig 2B and C). Hence, anatomic single-tunnel double-bundle-like effect, footprint enhancing ACL reconstruction is planned.

### Graft Preparation

An oblique incision 1 cm medial and distal to tibial tuberosity is taken. Careful subcutaneous dissection is done to the Sartorius fascia. Oblique incision is taken over sartorial fascia just inferior to gracilis tendon and in line with the hamstring tendons. The semitendinosus tendon is hooked out with the help of mister forceps, is freed from both vinculae, and is harvested with the help of a closed tendon striper. Similarly, gracilis tendon is harvested. Both grafts are prepared on graft master board after removing all fat and muscle tissue. Eightfold graft sized 9.75 mm is prepared using Ethibond number 5. The graft is kept soaked in vancomycin mixed with normal saline solution for further use.

### Tunnel Placement

**Fig 3:**
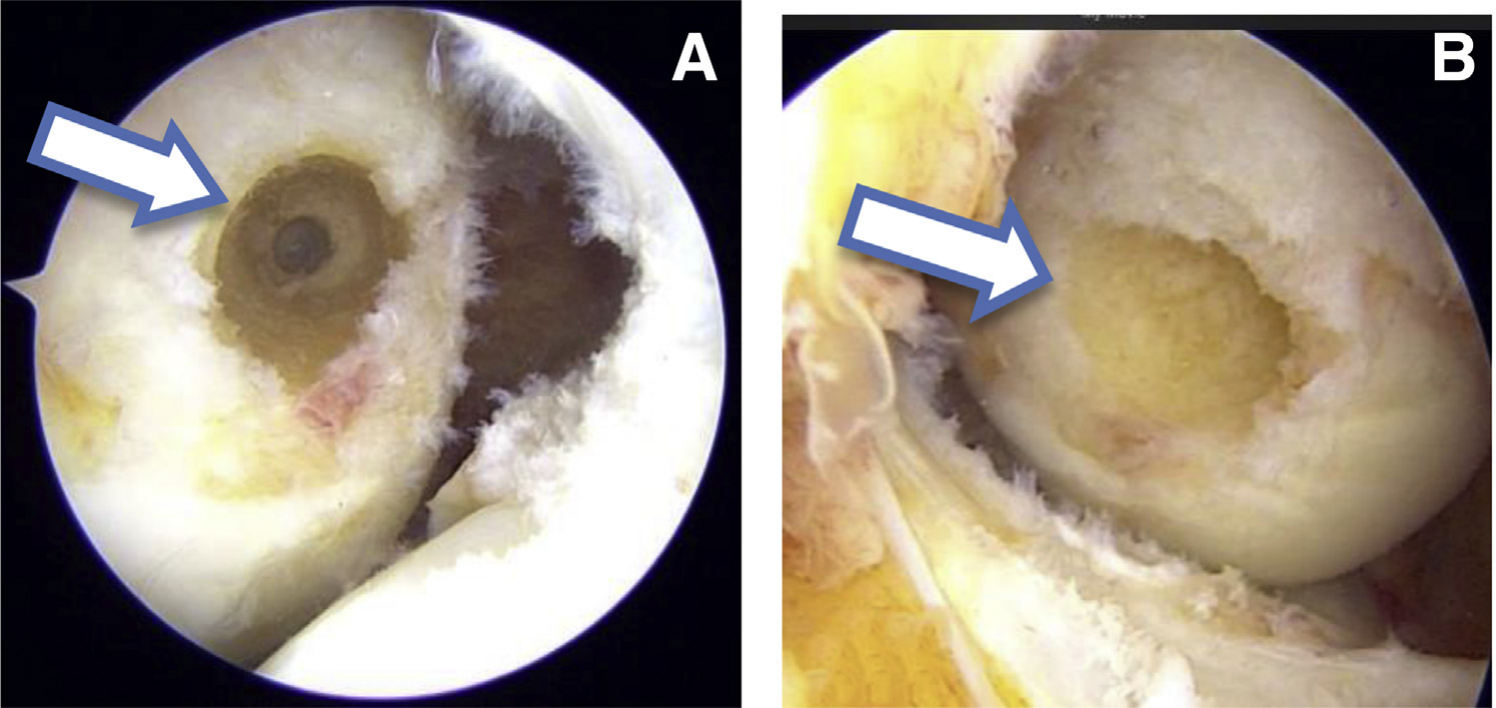
(A) Arthroscopic view from the transpatellar portal showing a circular tunnel in the oval femoral footprint as marked by an arrow. (B) Arthroscopic view from the posteromedial portal showing a circular tunnel in the oval femoral footprint as marked by an arrow.

**Fig 4:**
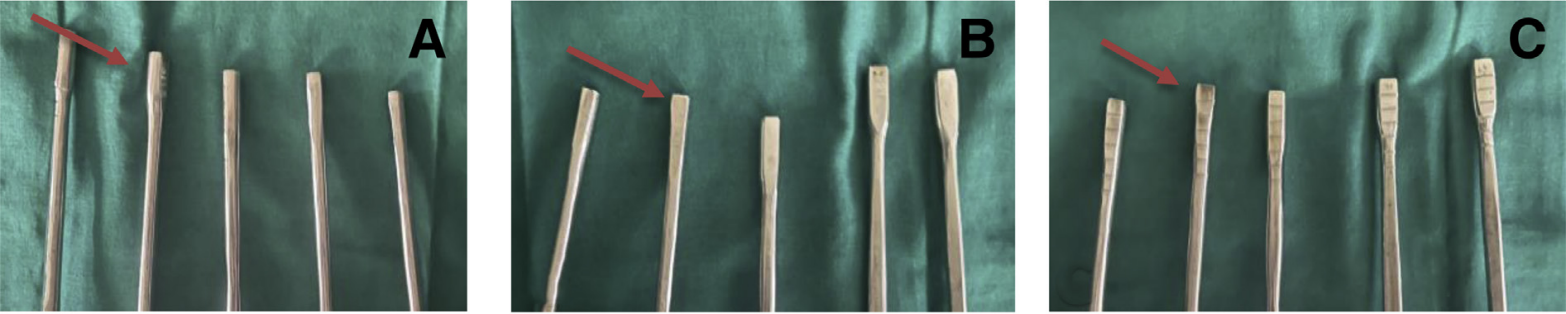
Our newly designed tunnel dilators of various sizes. (A) Side view, (B) back view, and (C) front view with markings at 5 mm interval as marked by red arrows in all 3 images.

**Fig 5:**
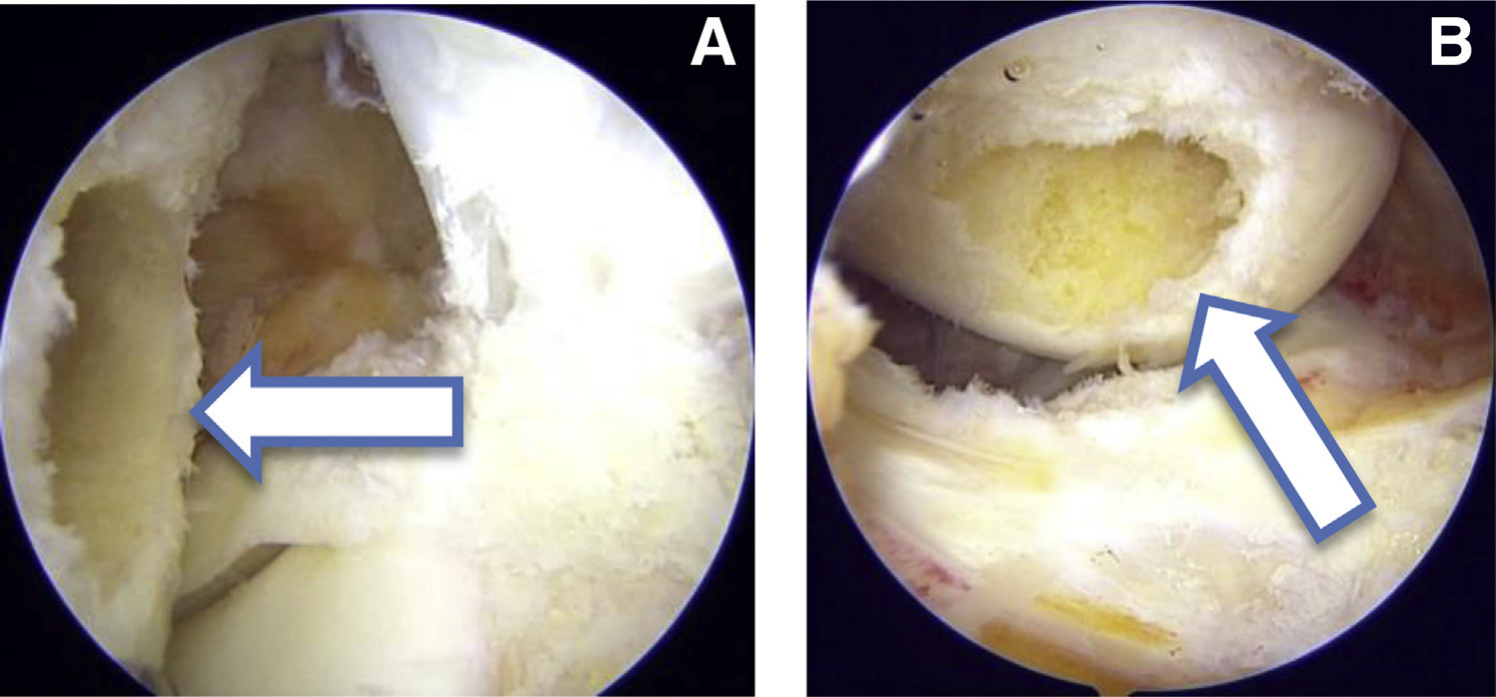
(A) Arthroscopic view from transpatellar portal showing an oval femoral tunnel after dilation with our newly designed tunnel dilators as marked by a solid arrow. (B) Arthroscopic view from the posteromedial portal also showing an oval tunnel after tunnel dilation with our newly designed tunnel dilators as marked by a solid arrow.

Viewing from TP portal first femoral tunnel is prepared through low AM portal. A 6 mm femoral offset guide (Stryker) is used to pass a guidewire from medial to lateral cortex of lateral femoral condyle. Then 4.5mm drill is used to prepare the tunnel throughout the length of lateral femoral condyle. Total length of the tunnel is 35 mm. Then an 8 mm drill is used to create a 20 mm socket. At this stage the view from TP and PM portal (Fig. 3) clearly showed a circular tunnel in an oval footprint. Hence, we used our newly designed oval tunnel dilators (Chetan Meditech Pvt. Ltd., Ahmedabad, India) to enhance the original footprint. These oval tunnel dilators comes in various sizes like 7 × 8 mm, 7 × 9 mm, 7 × 10 mm, 8 × 9 mm, 8 × 10 mm, 8 × 11 mm, 9 × 10 mm, 9 × 11 mm and further (Fig. 4). The rationale behind preparing this oval tunnel is to keep the anteroposterior distance same and to increase the superoinferior distance to enhance footprint coverage. So, to get maximum footprint coverage we have sequentially dilated the femoral tunnel using 8 × 9 mm and 8 × 10 mm dilators thereby increasing superoinferior coverage and keeping anteroposterior coverage same. After tunnel dilation view from TP and PM portal clearly shows an oblique femoral tunnel compared to circular femoral tunnel (Fig. 5). This view also shows enhancement of footprint coverage after tunnel dilatation by our specially designed oval tunnel dilators.

**Fig 6:**
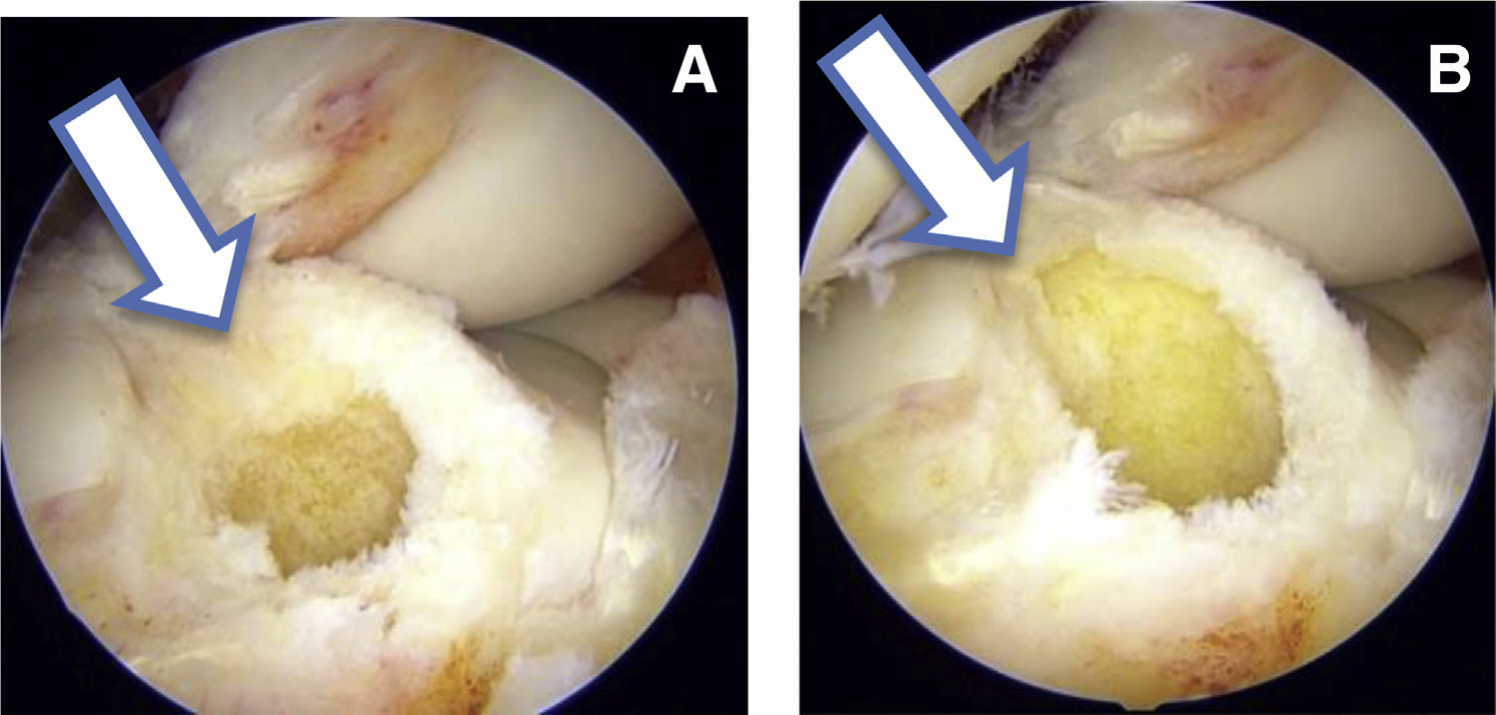
(A) Arthroscopic view from the anterolateral portal showing a circular tibial tunnel after drilling with an 8 mm drill bit as marked by a solid arrow. (B) Arthroscopic view from the anterolateral portal showing rectangular tibial tunnel after dilation using our designed tunnel dilators as marked by a solid arrow.

Next tibial tunnel is prepared by using tip aimer zig (Acufex; Smith & Nephew, London, UK). The tip of the zig is placed in the center of tibial footprint at an angle of 55°. Guidewire is drilled from the AM tibia into the center of tibial footprint. Furthermore, an 8 mm tunnel is created using the same size drill bit. View from high AL portal showed a circular tunnel in a rectangular footprint. So to enhance the footprint coverage we have used the same oval tunnel dilators which is used in femur, sized 8 × 9 mm and 8 × 10 mm. With this dilator the tibial tunnel is sequentially dilated, increasing anteroposterior coverage and maintaining mediolateral coverage. The view from the high AL portal shows how a circular tunnel is converted to a rectangular tunnel (Fig. 6) with our specially prepared oval tunnel dilators.

### Graft Passage and Fixation

**Fig 7:**
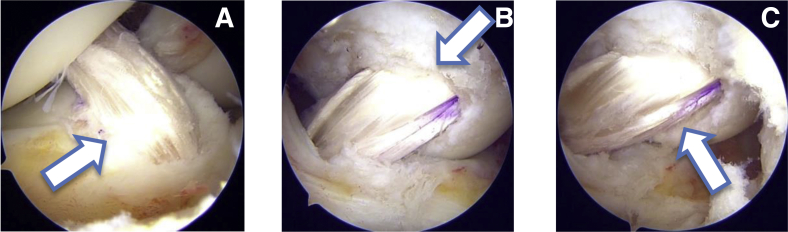
(A) Arthroscopic view from anterolateral portal (after graft fixation) showing complete rectangular tibial footprint coverage nearing to the original footprint as marked by a solid arrow. (B) Arthroscopic view from the posteromedial portal (after graft fixation) with the knee in 90° flexion showing near-full femoral footprint coverage as marked by solid arrow. (C) Arthroscopic view from the posteromedial portal (after graft fixation) with the knee in extension showing differential tension in the graft as marked by a solid arrow.

The graft diameter is 9 mm, and the tunnel dimension is 8 × 10 mm. As a soft tissue graft, it will squeeze from one side and will expand on other side. Hence, this circular graft was snugly fit in the rectangular tunnel. The graft is passed from the tibial to the femoral side and fixed with an adjustable loop (tightrope from Arthrex, Naples, FL) on the femoral side. On the tibial side the graft is fixed with a biocomposite 10 × 25 mm–sized screw (Arthrex). As cancellous bone, the circular screw has good purchase in this rectangular tunnel. Next a view from the high AL portal shows complete rectangular tibial footprint coverage nearing the original footprint (Fig. 7A). This view also shows PL bundle reconstruction as shown in video. View from low AM portal also shows a near-double-bundle reconstruction.

Similarly the view from the PM portal shows near-full femoral footprint coverage (Fig. 7 B and C). It also shows a tight AM bundle and a lax PL bundle in the extension and vice versa in flexion as shown in Video 1. A 10 mm drain is placed in the joint, and subcutaneous closure of portal is done.

**Fig 8:**
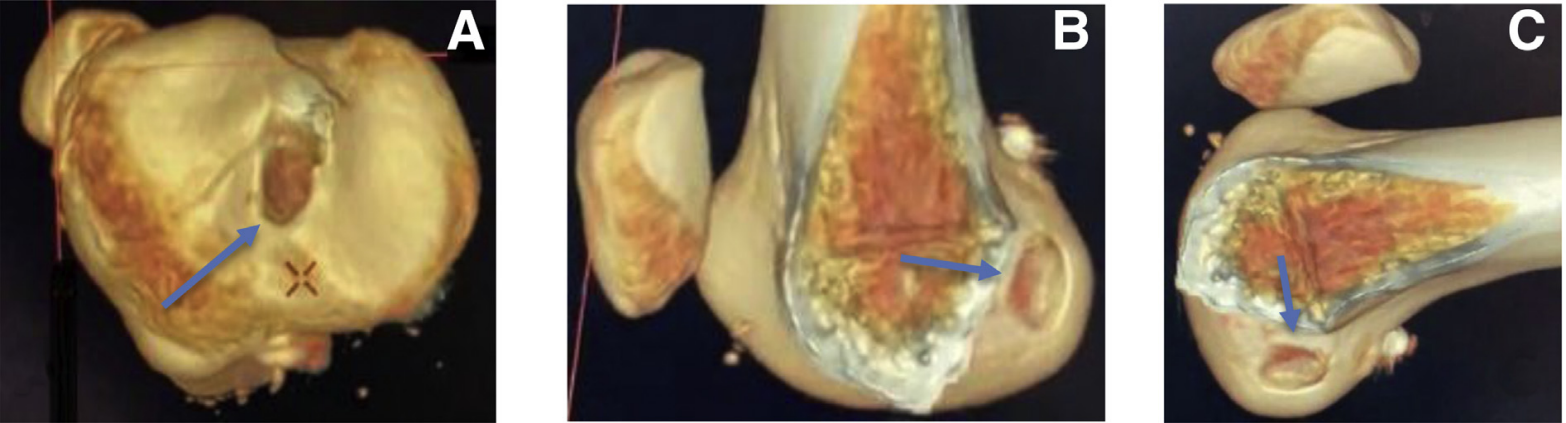
(A) Postoperative 3-dimensional computed tomography showing rectangular tibial tunnel. (B) Oval femoral tunnel in extension. (C) Oval femoral tunnel in flexion as marked by blue arrow in all 3 images.

Postoperative evaluation with computed tomography (CT) scanning is done to evaluate the postoperative footprint anatomy and tunnel position. A clear, near-anatomic oval reconstruction of the femoral and tibial footprint is seen on postoperative 3-dimensional (3D)-CT, which is comparable to intraoperative imaging (Fig. 8).

### Rehabilitation

Table 2 presents the postoperative rehabilitation process.

## Discussion

The ACL consists of the AM and PL bundle that works in synchronization during knee movements. The AM bundle is tight in flexion, and the PL bundle is tight in extension.^5^ Ideally both bundle reconstruction is needed for a good outcome of ACL reconstruction surgery. Muneta et al.^15^ introduced the double-bundle technique in which 2 tunnels were made in both the femur and the tibia to reconstruct both bundles and to increase footprint coverage. But it has its own disadvantages such as tunnel coalescence, longer surgical time, high technical expertise, high cost, and difficult revision surgery.^16^

Thus anatomic single-bundle ACL reconstruction became popular because it gave similar functional outcome in comparison to double-bundle reconstruction but had less rotational stability.^17^^,^^29^ There is increasing evidence that indicates that the anatomic reconstruction of AM and PL bundles will better restore normal knee kinematics, particularly internal and external rotation.^30^, ^31^, ^32^, ^33^, ^34^

The AM bundle is primarily responsible for stabilization of the knee in the anterior-posterior direction, whereas the PL bundle provides rotational stability.^1^^,^^4^ Our technique provides a double-bundle-like effect with a single oval/rectangular tunnel created by our specially designed oval tunnel dilators.

Therefore we assume the technique has more rotational stability compared to anatomic single-bundle reconstruction and further assume that the need for lateral extra-articular tenodesis or ALL reconstruction in primary ACL reconstruction may reduce with our technique.

Petersen et al.^35^ and Nakase et al.^36^ reported that they developed a technique of anatomic footprint reconstruction of the ACL with oval tunnels and rounded rectangle femoral dilators. Both of these techniques have enhanced the femoral side footprint with oval tunnels, but on the tibial side it was the same circular tunnel. Thus the graft in these studies would be oval at the femoral end and rounded at the tibial end. Our technique created oval tunnels at both the tibial and femoral side, thereby enhancing the footprint coverage on both sides. Wen et al.^37^ reported 4 theories to explain the advantage of the oval femoral tunnel technique. First, an oval femoral tunnel provides a larger surface area for a better blood supply from the adjacent cancellous bone surrounding the femoral tunnel. Second, an oval femoral tunnel closely resembles ACL anatomic insertions and restores natural ACL morphology. Third, the grafts used in an oval femoral tunnel should not easily rotate, which is advantageous for tendon bone healing. Fourth, use of the dilator technique, which ensures maximum preservation of the cancellous bone, leads to better mechanical stability.^37^ Zhao et al.^38^ reported that a flattened bone tunnel accelerated tendon bone healing in the early period after ACL reconstruction in a rabbit model. The same theories apply to our technique, not only on the femoral but also on the tibial side.

The footprint coverage in the single-bundle reconstruction technique is 57% of the native tibial insertion as mentioned by Kopf et al.^19^ and 61% of the femoral insertion as mentioned by Hensler et al.^20^ Siebold et al.^39^ reported that the femoral insertion was a long oval shape, and its size was 15 ± 3 mm × 8 ± 2 mm. Yasuda et al.^40^ reported that ACL femoral footprint is egg shaped. Ferretti et al.^24^ reported that the wide and long distance of femoral attachment was 17.2 ± 1.2 mm × 9.9 ± 0.8 mm. Mochizuki et al.^41^ and Luites et al.^42^ reported that the shape of the femoral attachment was an oval. Iwahashi et al.^43^ reported that the size of the oval-shaped femoral insertion was 17.4 ± 0.9 mm × 8 ± 0.5 mm. The tibial attachment of most anatomic studies was reported to be an average of 10 to 11 mm wide and 17 to 18 mm long, with an average area of 136 ± 33 mm^2^.^44^ Hence, it is clear that the femoral and tibial footprints are not circular but oval and rectangular in shape. Our technique recreates this oval femoral and rectangular tibial tunnel with our specially designed tunnel dilators. The study by Oshima et al.^45^ showed that the cross-sectional shape of the fourfold ST graft is not round but oval in shape, and the graft was well fitted in the rounded rectangular tunnel compared to the round tunnel. Our technique also uses the same graft with a rectangular tunnel. The graft failure rate is 7.2% if the ACL graft diameter is less than 8 mm^46^^,^^47^; this will be less likely with our technique because the graft size will always be greater than 8 mm

Our technique had some unique advantages and some disadvantages as mentioned in Table 1

**Table 1:** Advantages and Disadvantages of Our Technique

Advantages
Single oval/rectangular tunnel
More footprint coverage as compared to anatomical single-bundle reconstruction
Near-double-bundle-like effect
Cost-effective and less complicated as compared to double-bundle reconstruction
Easily reproducible
It will increase aperture healing area of graft
Less chance of failure because graft size will always be > 8 mm
Disadvantages
Extra transpatellar portal needed
Special tunnel dilators needed
Need to harvest both hamstrings

**Table 2:** Rehabilitation

1-2 weeks—static quadriceps exercises, heel slide 0° to 60°, active supine straight leg raising, ankle pump, and partial weightbearing walking with crutches
2-4 weeks—continue all exercises in addition to prone knee flexion, prone active straight leg raising, heel slide up to 120°, active knee extension, and isometric quadriceps and hamstring strengthening
4-6 weeks—continue all exercises in addition to full weightbearing walking, TheraBand strengthening for quadriceps and hamstrings, and heel slide up to full range of flexion
6-8 weeks—continue the above exercises in addition to starting half squats, cycling, gait training, and muscle strengthening with weights
8-12 weeks—single step at a time stair climbing and strengthening exercises
12 weeks onward—normal stair climbing, isotonic muscle strengthening, and gym training
6 months onward—slow jogging, proprioception training, and wobble board balancing
6-9 months—slow contact sports and cutting exercises
9 months onward—contact sports

This technique has a few limitations. It will be difficult to reconstruct 100% of the footprint with a single tunnel. Further biomechanical and clinical studies are required to support our double-bundle-like function and footprint enhancement by this technique.

In conclusion our technique of ACL reconstruction will provide more footprint coverage with a double-bundle-like effect using a single tunnel at both the femur and the tibial side. We believe it will provide more rotational stability compared to anatomic single-bundle reconstruction and fewer complications compared to the double-bundle reconstruction.

## Supplementary Data

ICMJE author disclosure forms

Video 1 Surgical details of our anterior cruciate ligament reconstruction technique.

## References

[1] Chhabra A., Starman J.S., Ferretti M. (2006). Anatomic, radiographic, biomechanical, and kinematic evaluation of the anterior cruciate ligament and its two functional bundles. J Bone Joint Surg Am.

[2] Gabriel M.T., Wong E.K., Woo S.L.-Y. (2004). Distribution of in situ forces in the anterior cruciate ligament in response to rotatory loads. J Orthop Res.

[3] Tischer T., Ronga M., Tsai A. (2009). Biomechanics of the goat three bundle anterior cruciate ligament. Knee Surg Sports Traumatol Arthrosc.

[4] Murray P.J., Alexander J.W., Gold J.E. (2010). Anatomic double- bundle anterior cruciate ligament reconstruction: Kinematics and knee flexion angle-graft tension relation. Arthroscopy.

[5] Petersen W., Zantop T. (2007). Anatomy of the anterior cruciate ligament with regard to its two bundles. Clin Orthop Relat Res.

[6] Nordenvall R., Bahmanyar S., Adami J., Stenros C., Wredmark T., Fellander-Tsai L. (2012). A population-based nationwide study of cruciate ligament injury in Sweden, 2001-2009: Incidence, treatment, and sex differences. Am J Sports Med.

[7] Webb J., Corry I. (2000). Epidemiology of knee injuries: diagnosis and triage. Br J Sports Med.

[8] Sanders T.L., Maradit Kremers H., Bryan A.J. (2016). Incidence of anterior cruciate ligament tears and reconstruction: A 21-year population-based study. Am J Sports Med.

[9] Gianotti S.M., Marshall S.W., Hume P.A., Bunt L. (2009). Incidence of anterior cruciate ligament injury and other knee ligament injuries: a national population-based study. J Sci Med Sport.

[10] Lemaire M. (1967). Ruptures anciennes du ligament croisé antérieur. Fréquence-clinique-traitement. J Chir.

[11] Lemaire M. (1975). Instabilité chronique du genou. Techniques et résultat des plasties ligamentaires en traumatologie sportive. J Chir.

[12] Insall J., Joseph D., Aglietti P., Campbell R. (1981). Bone-block iliotibial-band transfer for anterior cruciate insufficiency. J Bone Joint Surg Am.

[13] Tashman S., Collon D., Anderson K., Kolowich P., Anderst W. (2004). Abnormal rotational knee motion during running after anterior cruciate ligament reconstruction. Am J Sports Med.

[14] Tashman S., Kolowich P., Collon D., Anderson K., Anderst W. (2007). Dynamic function of the ACL-reconstructed knee during running. Clin Orthop Relat Res.

[15] Muneta T., Sekiya I., Yagishita K., Pguichi T., Yamamoto H., Shinomiya K. (1999). Two bundles reconstruction of the anterior cruciate ligament using semitendinosis tendon with endo buttons: Operative technique and preliminary results. Arthroscopy.

[16] Cameron M., Buchgraber A., Passler H. (1997). The natural history of the anterior cruciate ligament-deficient knee. Changes in synovial fluid cytokine and keratan sulfate concentrations. Am J Sports Med.

[17] Chen H., Chen B., Tie K. (2018). Single-bundle versus double-bundle autologous anterior cruciate ligament reconstruction: a meta-analysis of randomized controlled trials at 5-year minimum follow-up. J Orthop Surg Res.

[18] Chahla J., Menge T.J., Mitchell J.J., Dean C.S., LaPrade R.F. (2016). Anterolateral ligament reconstruction technique: An anatomic-based approach. Arthrosc Tech.

[19] Kopf S., Pombo M.W., Szczodry M., Irrgang J.J., Fu F.H. (2011). Size variability of the human anterior cruciate ligament insertion sites. Am J Sports Med.

[20] Hensler D., Working Z.M., Illingworth K.D., Thorhauer E.D., Tashman S., Fu F.H. (2011). Medial portal drilling: Effects on the femoral tunnel aperture morphology during anterior cruciate ligament reconstruction. J Bone Joint Surg Am.

[21] Siebold R. (2011). The concept of complete footprint restoration with guidelines for single- and double-bundle ACL reconstruction. Knee Surg Sports Traumatol Arthrosc.

[22] Colombet P., Robinson J., Christel P. (2006). Morphology of anterior cruciate ligament attachments for anatomic reconstruction: A cadaveric dissection and radiographic study. Arthroscopy.

[23] Duthon V.B., Barea C., Abrassart S., Fasel J.H., Fritschy D., Menetrey J. (2006). Anatomy of the anterior cruciate ligament. Knee Surg Sports Traumatol Arthrosc.

[24] Ferretti M., Ekdahl M., Shen W., Fu F.H. (2007). Osseous landmarks of the femoral attachment of the anterior cruciate ligament: An anatomic study. Arthroscopy.

[25] Markatos K., Kaseta M.K., Lallos S.N., Korres D.S., Efstathopoulos N. (2013). The anatomy of the ACL and its importance in ACL reconstruction. Eur J Orthop Surg Traumatol.

[26] Shino K., Suzuki T., Iwahashi T. (2010). The resident’s ridge as an arthroscopic landmark for anatomical femoral tunnel drilling in ACL reconstruction. Knee Surg Sports Traumatol Arthrosc.

[27] Zauleck M.K., Gabriel S., Fischmeister M.F., Hirtler L. (2014). Origin of the anterior cruciate ligament and the surrounding osseous landmarks of the femur. Clin Anat.

[28] Guenther D., Irarrázaval S., Nishizawa Y. (2017). Variation in the shape of the tibial insertion site of the anterior cruciate ligament: classification is required. Knee Surg Sports Traumatol Arthrosc.

[29] Komzák M., Hart R., Feranec M., Šmíd P., Kocová R. (2018). In vivo knee rotational stability 2 years after double-bundle and anatomic single-bundle ACL reconstruction. Eur J Trauma Emerg Surg.

[30] Aglietti P., Giron F., Cuomo P., Losco M., Mondanelli N. (2007). Single- and double-incision double-bundle ACL reconstruction. Clin Orthop Relat Res.

[31] Järvelä T. (2007). Double-bundle versus single-bundle anterior cruciate ligament reconstruction: A prospective, randomize clinical study. Knee Surg Sports Traumatol Arthrosc.

[32] Hussein M., van Eck C.F., Cretnik A., Dinevski D., Fu F.H. (2012). Prospective randomized clinical evaluation of conventional single-bundle, anatomic single-bundle, and anatomic double-bundle anterior cruciate ligament reconstruction: 281 cases with 3- to 5-year follow-up. Am J Sports Med.

[33] Muneta T., Koga H., Mochizuki T. (2007). A prospective randomized study of 4-strand semitendinosus tendon anterior cruciate ligament reconstruction comparing single-bundle and double-bundle techniques. Arthroscopy.

[34] Yasuda K., Kondo E., Ichiyama H., Tanabe Y., Tohyama H. (2006). Clinical evaluation of anatomic double-bundle anterior cruciate ligament reconstruction procedure using hamstring tendon grafts: Comparisons among 3 different procedures. Arthroscopy.

[35] Petersen W., Forkel P., Achtnich A., Metzlaff S., Zantop T. (2013). Technique of anatomical foot-print reconstruction of the ACL with oval tunnels and medial portal aimers. Arch Orthop Trauma Surg.

[36] Nakase J., Takata Y., Shimozaki K. (2021). Clinical study of anatomical ACL reconstruction using a rounded rectangular dilator. BMC Musculoskelet Disord.

[37] Wen Z., Zhang H., Yan W. (2020). Oval femoral tunnel technique is superior to the conventional round femoral tunnel technique using the hamstring tendon in anatomical anterior cruciate ligament reconstruction. Knee Surg Sports Traumatol Arthrosc.

[38] Zhao F., Hu X., Zhang J. (2019). A more flattened bone tunnel has a positive effect on tendon-bone healing in the early period after ACL reconstruction. Knee Surg Sports Traumatol Arthrosc.

[39] Siebold R., Ellert T., Metz S., Metz J. (2008). Femoral insertions of the anteromedial and posterolateral bundles of the anterior cruciate ligament: Morphometry and arthroscopic orientation models for double-bundle bone tunnel placement—A cadaver study. Arthroscopy.

[40] Yasuda K., Kondo E., Ichiyama H. (2004). Anatomic reconstruction of the anteromedial and posterolateral bundles of the anterior cruciate ligament using hamstring tendon grafts. Arthroscopy.

[41] Mochizuki T., Muneta T., Nagase T., Shirasawa S., Akita K.I., Sekiya I. (2006). Cadaveric knee observation study for describing anatomic femoral tunnel placement for two-bundle anterior cruciate ligament reconstruction. Arthroscopy.

[42] Luites J.W., Wymenga A.B., Blankevoort L., Kooloos J.G. (2007). Description of the attachment geometry of the anteromedial and posterolateral bundles of the ACL from arthroscopic perspective for anatomical tunnel placement. Knee Surg Sports Traumatol Arthrosc.

[43] Iwahashi T., Shino K., Nakata K. (2010). Direct anterior cruciate ligament insertion to the femur assessed by histology and 3-dimensional volume-rendered computed tomography. Arthroscopy.

[44] Liu Z., Hu X., Zhang X., Jiang Y., Wang J., Ao Y. (2018). Clinical study of anatomical ACL reconstruction with adjustable oval shaped bone tunnels: A CT evaluation. Am J Transl Res.

[45] Oshima T., Nakase J., Numata H. (2016). The cross-sectional shape of the fourfold semitendinosus tendon is oval, not round. J Exp Orthop.

[46] Alkhalaf F.N.A., Hanna S., Alkhaldi M.S.H., Alenezi F., Khaja A. (2021). Autograft diameter in ACL reconstruction: Size does matter. SICOT J.

[47] Alomar A.Z., Nasser A.S.B., Kumar A., Kumar M., Das S., Mittal S. Hamstring graft diameter above 7 mm has a lower risk of failure following anterior cruciate ligament reconstruction [published online February 23, 2021]. Knee Surg Sports Traumatol Arthrosc. 10.1007/s00167-021-06503-0.

